# Revisiting Chagas' disease diagnostic strategies in light of different scenarios of *Trypanosoma cruzi* infection

**DOI:** 10.1590/0074-02760210444chgsb

**Published:** 2022-06-01

**Authors:** Constança Carvalho Britto

**Affiliations:** 1 Fundação Oswaldo Cruz-Fiocruz, Laboratório de Biologia Molecular e Doenças Endêmicas

Chagas disease (CD) affects millions of people, mostly poor and neglected populations residing in endemic countries in Latin America. Diagnosing CD is not simple and results in limited access to treatment. Considering the complexity of the disease attributed to factors as, different routes of infection by *Trypanosoma cruzi*, high genetic heterogeneity of the parasite, the need to use different detection methods associated to the specific stages of the disease and patterns of transmission, Schijman and colleagues, with great propriety and clarity, revisit the accuracy of CD diagnosis in different existing scenarios in endemic and non-endemic areas. A broad panel of diagnostic strategies and their application is presented, ranging from conventional parasitological techniques to more complex serological and molecular assays. Due to the particularities of each test, the authors present specific diagnostic algorithms to be applied for early *T. cruzi* detection in the acute phase of CD, to assess congenital transmission through the screening of pregnant women and their offspring, epidemiological surveys, surveillance for vector-borne transmission, blood bank and organ donors screening and for the diagnosis of chronic CD. The tests used for the diagnosis of CD should be universal, *i*.*e*., capable of detecting all human-infecting parasite lineages. In addition to laboratory tests, the epidemiology and clinical context are pertinent for the interpretation of results.

Methods to prevent the increase in the incidence of the disease are vector control, control of blood banks, and carrying out tests on pregnant women to prevent congenital transmission.^1^ However, the challenges are amplified in the face of estimates in which more than 80% of people affected by CD do not have access to diagnosis or treatment, which supports the high impact of morbidity and mortality and the association with social cost. Moreover, the rate of mother-to-child transmission remains unchanged, gaining greater evidence in endemic countries where measures to control vector and transfusion transmission were adopted; besides being the main source of new cases in non-endemic countries, such as Spain, which recommends systematic screening for CD in Latin American pregnant women.^2^

In the initial phase of infection, circulating parasites can be detected by direct microscopic observation. Direct testing with fresh blood is more sensitive than stained smear. If trypomastigotes are not observed in the blood of patients suspected of having acute CD, concentration tests (microhematocrit or Strout) should be carried out to increase sensitivity. The sensitivity of microscopic observation is highly dependent on the operator. In suspected cases of congenital transmission, it is important to test for the mother’s infection. Around 60% of congenitally infected newborns are asymptomatic at birth. In this review, the authors highlight the importance of preventing vertical transmission and the need to early diagnose and treat congenitally infected newborns, in view of the high rate of cure when treatment is administered close to birth.^3^ A detailed up-to-date diagnostic algorithm for mother-to-child infection is presented, combining direct parasitological and/or molecular assays within 72 h after birth and serology over a 9-12 months period.

The diagnosis of *T*. *cruzi* infection becomes more difficult during the chronic phase, in which, the low and intermittent parasitaemia leads to the need for diagnostic tools with higher sensitivity. In this phase, characterised by high levels of anti-*T*. *cruzi* IgG, the diagnosis is essentially serological but its results can turn difficult to interpret, and the parallel use of at least two different tests is strongly advised. Given this scenario, the discovery of new diagnostic biomarkers has been a challenge to improve accuracy and, thus, reduce the number of inconclusive serological results. Nowadays, most commercial tests use recombinant antigens, synthetic peptides or antigenic matrices based on chimeric *T*. *cruzi* proteins. To avoid interference on the diagnostic test performance across the Americas, these antigens should be conserved and covering the high diversity of parasite lineages. It is recognised that the antigenic diversity of *T*. *cruzi* is one important factor leading to a reduced concordance among different commercial tests applied in distinct environments. Here, Schijman and colleagues also discuss the use of rapid diagnostic tests (RDTs), as effective alternatives for large-scale screening and point-of-care (POC) diagnosis of chronically infected individuals residing in resource limited areas in different CD epidemiological scenarios. Fast results, easy handling, high sensitivity/specificity of new developed RDTs are key parameters for their use in healthcare facilities or in remote areas.^4^

PCR-based assays have proven useful during acute-phase or chronic-phase reactivations due to higher sensitivity compared to parasitological methods. However, molecular detection of *T*. *cruzi* in the chronic phase gives positive results in 40-70% of the seropositive patients; so, these methods have a limited diagnosis value in chronic CD patients. The variable sensitivity depends on the level of parasitaemia, sample volume, DNA extraction method, the selected PCR target, the characteristics of the study populations and the genetic diversity of parasite’s lineages. A negative PCR result does not exclude infection and a positive result confirms the presence of the parasite and can be associated to therapeutic failure of anti-parasitic treatments and parasite reactivation in immunosuppressed patients.^5^ ^,^ ^6^ By using PCR-based assays it is possible to molecular characterise *T*. *cruzi* genotypes directly from the blood of infected individuals.^7^ Quantitative real-time PCR (qPCR) was developed allowing the simultaneous detection and quantification of the parasite’s DNA. However, the in-house developed qPCR protocols showed variable levels of analytical reliability, specificity and sensitivity, restricting their use in the clinical practice. Nowadays, commercially available tests have been introduced, turning possible the standardisation of protocols between laboratories. In the present review, the authors show in Table II the description of primers and probes sequences for *T*. *cruzi* detection/quantification that were previously validated for use in the in-house TaqMan qPCR protocols. Up to date there are three commercial qPCR kits targeting the *T*. *cruzi* satellite DNA and one solely developed for the minicircles DNA that make up the parasite kDNA network.

Loop-mediated isothermal amplification (LAMP) methodology has been developed as an alternative approach for molecular diagnosis, easy to use in field conditions in areas with scarce resources. The inherent characteristics of LAMP (simpler, faster and cheaper than PCR-based assays), have attracted experts working in CD for the development of commercial kits with great potential application in endemic areas.^8^ ^,^ ^9^ The *Trypanosoma cruzi*-LAMP technology has been assayed in distinct scenarios of acute CD, as presented by the authors in Table I, showing high agreement with the real-time PCR results. If one considers the urgent need for improving the access to diagnosis (and treatment), mainly in CD endemic regions with scarce and resource-limited laboratories, the use of POC diagnostics would expand access to a larger number of patients. In this context, as stated by Schijman and colleagues, the LAMP technology, due to its own peculiarities could help fill these diagnostic gaps along with easier, faster and less costly platforms for DNA extraction.

As general conclusion of this important and attractive review, the authors highlight the urgent need for an increased access to and demand for effective diagnostics by improving the performance of diagnostic algorithms and methods, making them more available to poor and vulnerable populations in endemic countries in Latin America, in order to an effective control of CD.

Comments on the article: Schijman AG, Alonso-Padilla J, Longhi SA, Picado A. Parasitological, serological and molecular diagnosis of acute and chronic Chagas disease: from field to laboratory. Mem Inst Oswaldo Cruz. 2022; 117: e200444.
